# Non-Contact Respiration Measurement during Exercise Tolerance Test by Using Kinect Sensor

**DOI:** 10.3390/sports6010023

**Published:** 2018-03-13

**Authors:** Hirooki Aoki, Hidetoshi Nakamura

**Affiliations:** 1 Chitose Institute of Technology, Chitose 066-8655, Japan; 2 Department of Respiratory Medicine, Saitama Medical University, Iruma-gun 350-0495, Japan; hnakamur@saitama-med.ac.jp

**Keywords:** non-contact respiration measurement, motion capture, Kinect sensor

## Abstract

This study aims to assess non-contact respiration measurement during the exercise stress test using an upright bicycle ergometer and to evaluate the ventilation threshold value. We propose the tracking of the chest and abdomen by applying the motion capture function of the Kinect V2 sensor to cope with an increase in physical exercise accompanied by an increase in exercise intensity. In the proposed method, the region enclosed by the four joints corresponding to the left and right shoulders and the right and left hip extracted using the Kinect sensor is set as the region of interest. The region is updated in response to changes in body movements. By extracting the signal of the pedaling frequency component from the time series data of the volume in the region, only the volume change due to respiration was extracted. The point at which the increased rate of the volume change elevates is estimated as the ventilation threshold. The assessment of the efficacy of the proposed method by comparative analysis using an expiration gas analyzer confirmed that non-contact respiration evaluation is possible with an exercise intensity of about 160 W. Furthermore, the ventilation threshold estimated by the proposed method is ±10 W of the estimated value by expiratory gas analyzer.

## 1. Introduction

In rehabilitation institutes, the anaerobic threshold is typically evaluated by the exercise tolerance test to assess the systemic aerobic capacity [1]. Although an expiration gas analyzer is conventionally used for the assessment of the anaerobic threshold (AT), it is expensive. In addition, convenient assessment is difficult while using the expiration gas analyzer, because it requires mounting a gas mask on the face of participants, which poses significant problems, particularly in children and the elderly who usually have dfficulty wearing a mask. Thus, the development of a simple and cost-effective method to assess the AT is imperative for performing safe and effective exercise. 

The VT, an equivalent index of the AT, is evaluated from the change in minute ventilation (VE) obtained by the ramp load test using a bicycle ergometer [2]. The generation of anaerobic energy stimulates the production of the lactic acid, which when buffered with bicarbonate ion increases the ventilation volume by producing carbon dioxide [3]. Caiozzeo [4] demonstrated that the VT could be evaluated not only from the increased amount of carbon dioxide in expiration but also from the increased VE in the ramp load test. 

Previously, we investigated a non-contact measurement with respiration in the rest position by using pattern light projection [5], which elucidated that the volume change in the chest significantly correlated with the change in ventilation. In addition, this method established non-contact respiration measurement during pedaling with a recumbent bicycle ergometer [2]. The application of Caiozzeo’s VT calculation by VE elucidated that the VT could be calculated even by the non-contact respiration measurement [6]. In this method, the increase in the volume change because of the increase in the exercise intensity in the ramp load test is evaluated in the non-contact measurement, and the point at which the volume change rate increases is estimated as the VT, also referred as QVT. The result of this method suggests the feasibility of the systemic aerobic capacity assessment using the non-contact respiration measurement.

In our previous study mentioned above, although we used a recumbent bicycle ergometer, upright bicycle ergometers are more prevalent [7]. Hence, improving the versatility of the proposed method necessitates non-contact respiration measurement using an upright bicycle ergometer. In upright bicycle ergometers, the body motion by pedaling is higher than those in the recumbent types. However, non-contact respiration measurement by pedaling using an upright bicycle ergometer is considered challenging. Hence, one study proposed a method to facilitate stable non-contact respiration measurement by reducing the impact of the pedaling motion [8].

In our previous studies, we used a sensor based on the active stereo method, called fiber-grating vision sensor (FG sensor), which was developed independently by us and is not sold in general [9]. In 2010, Microsoft launched the Kinect sensor as a non-contact controller of the game console. The Kinect sensor is a three-dimensional vision sensor based on the active stereo in which the pattern light—called ‘light coding’—is projected onto an object, and the three-dimensional shape of the object is reconstructed by image analysis of the pattern light distribution. The Kinect sensor is cost-effective and it facilitates real-time processing of three-dimensional data measured by our own program because it supports USB connection to a PC. For this reason, Kinect sensors are used in a wide range of research fields for various purposes.

In another study of ours, we investigated the respiration measurement using the Kinect sensor as an alternative to the FG sensor in the ramp load test. The findings revealed that the quantitative respiration measurement in the resting posture is possible with the Kinect but not the FG sensor [10]. As mentioned above, since the change in the posture by pedaling is significant in the test using the upright bicycle ergometer, reducing the impact of the body movement on the assessment of signal is essential. Hence, we proposed a method to consider the part of the body (i.e., chest and abdomen) where the respiratory motion was likely to appear as a region of interest (ROI). Consequently, we illustrated that the accuracy of the quasi tidal volume (QTV) fluctuation can be enhanced by filtering the three-dimensional shape change in the ROI [11]. However, even this method did not facilitate the complete elimination of the effect of a large change in the posture and was, thus, considered difficult to apply at a high exercise intensity of ≥100 W. 

In 2014, a new version of the Kinect sensor (Kinect v2 sensor) was released, in which the measurement method was revised from active stereo to time-of-flight (TOF). The principle of TOF states that the distance is calculated from the time until the light projected from the light source is reflected by an object and reaches a light-receiving sensor. Typically, light-receiving sensors are distributed in a two-dimensional array and the distance distribution, in which the distance image can be acquired. Apparently, data obtained from the distance image facilitates three-dimensional shape reconstruction of the object. Although the measurement accuracy of the Kinect v2 sensor is almost equal to that of the previous version, its motion capture function has been enhanced for its easy use. 

By tracking the thoracoabdominal activity using the motion capture function of the Kinect v2 sensor, this study aims to propose a non-contact respiration measurement during the exercise tolerance test corresponding to an increase in the body movement with an increasing exercise intensity. Additionally, it assesses the validity of the proposed method by comparative experiment using the expiratory gas analyzer.

## 2. Materials and Methods

### 2.1. Configuration of the Measurement System

**Figure 1:**
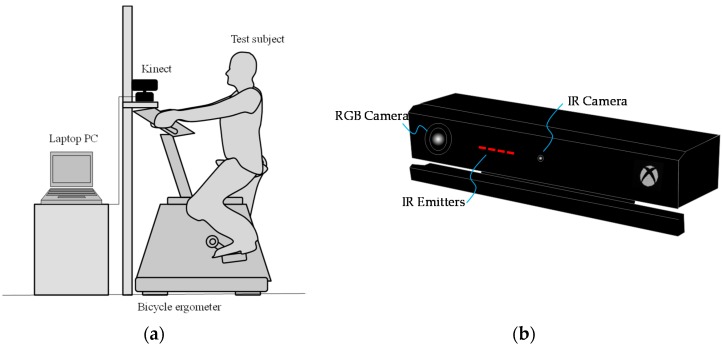
(**a**) System configuration and (**b**) Kinect v2 sensor.

Figure 1a shows the measurement system in this study. The Kinect sensor (Kinect v2 sensor, Microsoft Corporation, Redmond, WA, USA) is placed facing the test subject sitting on the saddle of the upright bicycle ergometer. The test subject is asked to pedal the bicycle at a constant rotation frequency, which was set at 1 Hz.

As described earlier, the Kinect v2 sensor can acquire the distance image by the TOF method, which is more recently known as a depth image. The Kinect v2 sensor is connected to the general-purpose laptop PC with a USB port. Then, a sequence of depth image obtained by the Kinect v2 sensor is processed by the PC. The Kinect v2 sensor primarily comprises a color camera (RGB camera), an infrared camera (IR camera), and an infrared projector (IR projector), as shown in Figure 1b [12]. Table 1 shows the specification of the Kinect. The field of view is 60° vertical and 70° horizontal. In addition, the resolution of the RGB image obtained by the RGB camera was 1080 p (1920 × 1080 pixels). In contrast, the resolution of the depth image obtained by the infrared depth sensor, comprising the IR camera and the IR projector, was 512 × 424 pixels. Notably, each pixel of the depth image has 13-bit information, and the depth resolution is 1 mm; in the depth image measurement, the limit depth range is 0.5–4.5 m.

**Figure 2:**
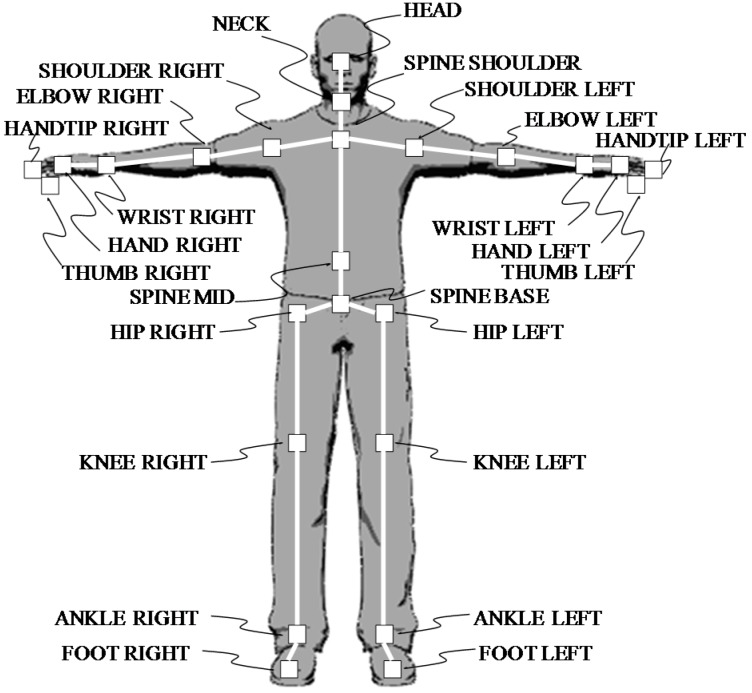
The 25 joints extracted by the markerless motion capture function of the Kinect v2 sensor.

The Kinect v2 sensor is characterized by a markerless motion capture function (also called skeleton tracking), which can estimate and track the coordinates of 25 joints of the human body (Figure 2).

### 2.2. Non-Contact Respiration Measurement

In this study, we measured breathing of participants who pedaled on the upright bicycle ergometer without contact in four steps as explained below.

#### 2.2.1. Setting of the ROI

**Figure 3:**
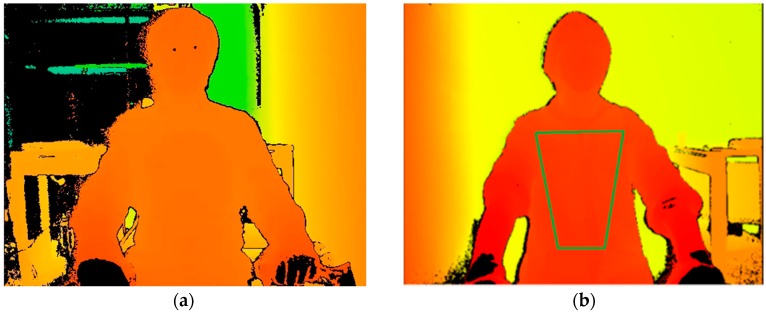
(**a**) Depth image and (**b**) setting of ROI (region of interest).

In the measurement system, the horizontal distance between the Kinect v2 sensor and the saddle tip of the bicycle ergometer was 0.7 m. In the depth image acquired by the Kinect v2 sensor, we imaged the upper body of subjects (Figure 3a). Then, using the joint extraction function of the Kinect v2 sensor, we extracted four joints as follows: right shoulder, left shoulder, right hip, and left hip. A region surrounded by these four joints was set as the ROI (Figure 3b). We anticipated changes in the body surface because of respiration because the body surface corresponding to the ROI was the chest wall. Since the coordinates of the four joints were shifted by the body movement, the ROI was updated in every frame. By using the depth information from the ROI, we performed respiration assessment by the following procedure.

#### 2.2.2. Volume Calculation

Based on the principle of triangulation, the depth of each pixel of the depth image can be converted to the three-dimensional coordinates of the vertex (*X*, *Y*, and *Z*) by the Equation (1)

(1) $$\begin{array}{l} X=\frac{\left(x_{p}-p_{h}/2)\tan(\theta_{h}/2\right)}{p_{h}/2}z_{p}\\ Y=\frac{\left(p_{v}/2-y_{p})\tan(\theta_{v}/2\right)}{p_{v}/2}z_{p}\\ Z=z_{p} \end{array}$$

where *x_p_* is the horizontal pixel coordinate on the depth image, *y_p_* is the vertical pixel coordinate on the depth image, *z_p_* is the depth value at a pixel coordinate (*x_p_* and *y_p_*), *p_h_* is the total pixel number of horizontal direction, *p_v_* is the total pixel number of vertical direction, *θ_h_* is the horizontal angle of view of the IR camera, and *θ_v_* is the horizontal angle of view of the IR camera. We resampled the coordinates of the vertices by applying the Delaunay triangulation with linear interpolation [13] and calculated the volume of the body surface in the ROI. 

#### 2.2.3. Extraction of the Respiration Component

**Figure 4:**
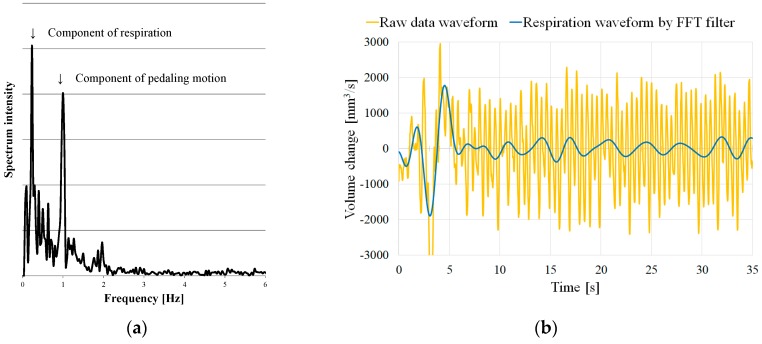
(**a**) Frequency spectrum of the measurement waveform and (**b**) extracted respiration waveform.

Next, we calculated the change in the volume in the ROI between frames. Changes in the volume were primarily caused by the pedal motion of subjects. In addition, the volume change slightly contained the component of the motion of the chest–abdominal wall caused by respiration. In the exercise tolerance test, subjects were asked to pedal at a constant rotation frequency, which, in this study, was set at 1 Hz (60 rpm). Figure 4a shows the frequency spectrum of the volume change. Two peaks emerged at a 1-Hz neighborhood and at lower-frequency band than 1 Hz. The peak at about 1 Hz is attributed to the pedaling motion of subjects. Here, we term the frequency reflected by the peak as ‘pedaling motion frequency’. However, as the peak at a lower frequency band than 1 Hz was caused by the respiratory motion, it was termed ‘respiration frequency’.

**Figure 5:**
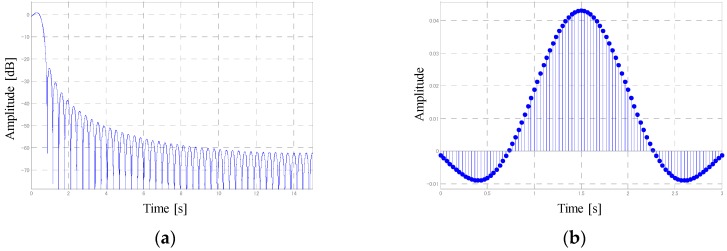
(**a**) Amplitude response and (**b**) impulse response of the designed digital filter.

Figure 4b shows that the respiration waveform was extracted by applying the FFT bandpass filter to the waveform of the volume change between frames. In this study, the bandwidth of the FFT bandpass filter was set between 0.1 and 0.7 Hz. Notably, it is essential to apply the FFT filter after the end of the measurement, and it is not possible to extract the breathing waveform in real time. Hence, real-time processing of the breathing waveform extraction was attained by applying a digital filter. The designed digital filter comprised a least-squares linear-phase FIR filter of the order 90. Figure 5 shows the characteristics of the digital filter.

#### 2.2.4. Calculation of the QTV

The respiration waveform calculated above represents the volume change between frames of the ROI. In the extracted respiratory waveform, the positive and negative are reversed between exhalation and inhalation, thereby allowing the separation of exhalation and inspiration by detecting the zero-crossing of the waveform. Integrating the area of the waveform of one expiration or one inspiration facilitates calculating the amount equivalent to the TV, defined as QTV; the QTV is not equal as the absolute amount of TV, but the rate of change in the QTV with time is equal to that of the TV. We defined QTV multiplied by breathing rate (number of breaths per minute) as quasi VE (QVE).

### 2.3. Participants

**Figure 6:**
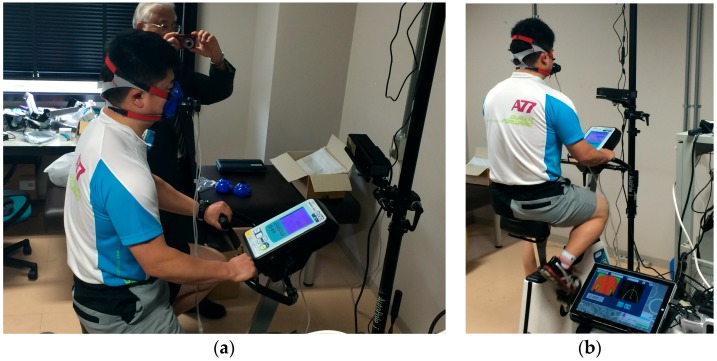
A subject pedaling an upright bicycle ergometer. (**a**) Side direction; (**b**) Back direction.

The validity of the method explained above was determined through experiments in this study. We conducted non-contact respiratory measurements during the exercise tolerance test on six male subjects (Subject A, B, C, D, E, and F). During the assessment, all subjects were asked to wear a T-shirt that remained in in close contact with their body. Wrinkles and twists of the material cloth might affect the measured value; however, since there are no means to validate this assumption, we did not consider its impact in this experiment. Figure 6 shows a subject pedaling the bicycle ergometer. This study was approved by the Institutional Ethical Review Board of our university (approval code: 13-044 (12 September 2013)), and all experiments were conducted in accordance with the Declaration of Helsinki. We obtained written informed consent from all participants after explaining the experimental procedures, risks, and benefits.

### 2.4. Procedures

The validity of the assessment using an upright bicycle ergometer (75XLII; Konami Sports Life Co., Ltd., Tokyo, Japan) was examined by comparing with the simultaneous assessment using an expiration gas analyzer (Aeromonitor AE-280S; Minato Medical Science Co., Ltd., Tokyo, Japan). The ergometric intensity was then increased automatically to 20 W/min ramp load from 0 W. All subjects continued pedaling for 8 min. However, if the fatigue reached the limit, exercise was stopped halfway. Furthermore, the examinee pedaling rate was guided by a metronome sound to keep around 60 rpm.

## 3. Results

**Figure 7:**
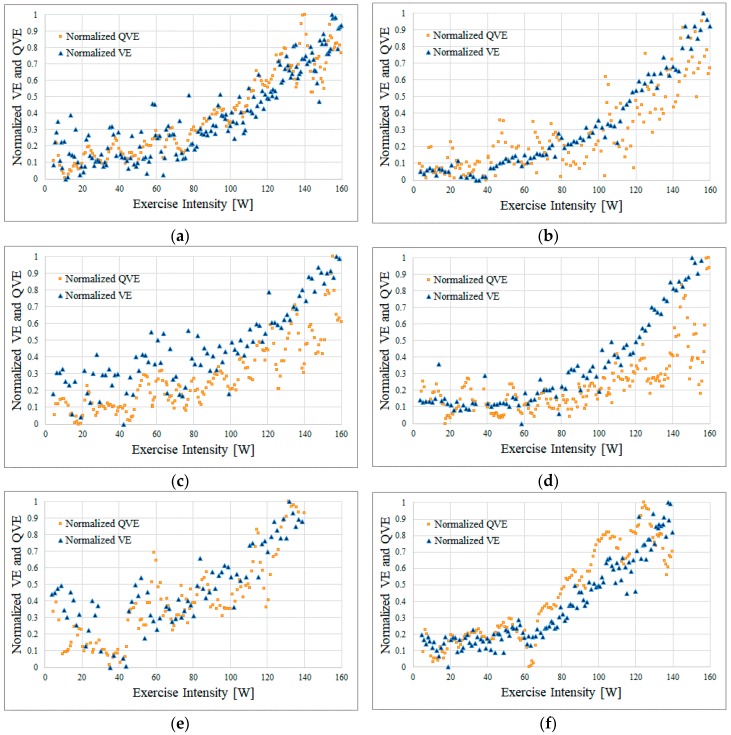
Results of the simultaneous measurement with the proposed method and expiratory gas analyzer. (**a**) Subject A; (**b**) Subject B; (**c**) Subject C; (**d**) Subject D; (**e**) Subject E; and (**f**) Subject F.

Figure 7 shows the results of the simultaneous measurement as follows: yellow squares, the QVE obtained by the proposed method; blue triangles, the VE obtained by the expiration gas analyzer; the horizontal axis, the normalized value of QVE/VE. With the minimum and the maximum as a standard, we calculated the normalized value so that the value changed from 0 to 1.

Among the six subjects, Subjects A, B, C, and D rode the bicycling ergometer up to an exercise intensity of 160 W. In contrast, Subjects E and F were unable to continue pedaling with the exercise intensity around 140 W because of fatigue and stopped exercising at 140 W.

**Figure 8:**
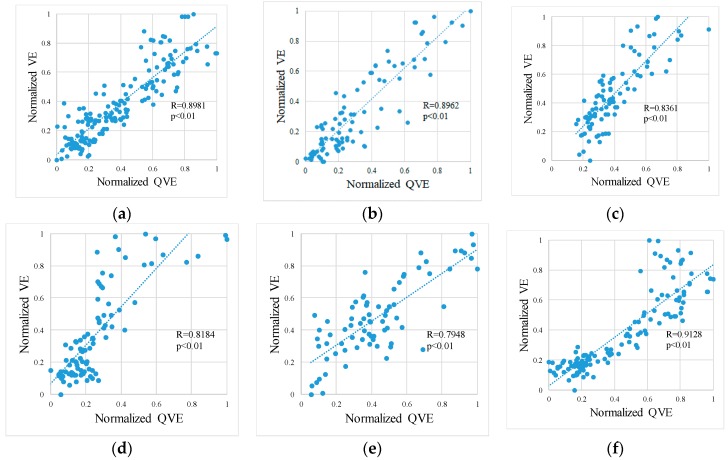
Scatter plots of VE and the QVE. (**a**) Subject A; (**b**) Subject B; (**c**) Subject C; (**d**) Subject D; (**e**) Subject E; and (**f**) Subject F.

In all results, compared to the VE, the QVE was highly variable (Figure 7). However, we observed that the VE and QVE changed with a similar tendency in each subject. Figure 8 shows scatter diagrams of the VE and QVE. Furthermore, the linear regression analysis revealed that the correlation coefficient was ≥0.79 in any of our subjects. For each subject, the bias and the 95% confidence interval (95% CI) of the difference between VE and QVE as well as VT estimated by the expiration gas analyzer and QVT estimated by the method proposed in our previous works [6] are shown in Table 2.

## 4. Discussion

This study suggests that non-contact respiration measurement can be performed even during pedaling movements with an exercise intensity of 140–160 W per the proposed method. In the method proposed previously [10], non-contact respiration measurement at an exercise intensity of ≥100 W was difficult; however, the proposed method in this study overcomes those limitations and proves its validity and usefulness.

**Figure 9:**
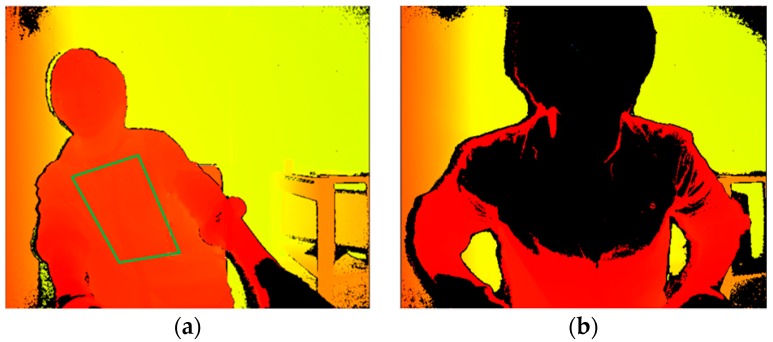
(**a**) Lateral bending position and (**b**) forward-bending position.

However, as the exercise intensity increased, we observed the tendency for the VE and QVE to diverge, which may warrant further investigation. For example, with Subject D, the VE and QVE started diverging at an exercise intensity of ≥120 W. At this time, Subject D was in a posture in which the left and right inclinations were significant (Figure 9a). Although the ROI was correctly set at this time, the large left and right movements presumably hindered the detection of respiration, which is moving in the depth direction. In addition, the ROI could not be set correctly because the subject was too close to the sensor, which restricted the precise respiration measurement (Figure 9b). This may be attributed to the fact that distance information is not measured because the upper body was set at a position closer than the lower limit (50 cm) of the sensing range of the Kinect v2. Thus, further study is necessary to elucidate the conditions at which the accuracy of non-contact respiration measurement deteriorates. As a countermeasure, two approaches are conceivable. The first approach is to set a longer distance between the subject and the sensor. Another approach is to place the backrest on the ergometer’s seat chair. When subjects pedal with their back in contact with the backrest, it is possible to prevent measurement from being impossible due to a forward-tilting posture.

The correlation coefficient between the VE and QVE in this study was ≥0.79 (Figure 8). An investigation by optoelectronic plethysmography (OEP) is a well-known study on the correlation between the volume change in the chest and exhalation flow during exercise. Apparently, OEP is an application of motion capture using markers that enables strict volume measurement than markerless motion capture. A previous study on OEP revealed that the correlation coefficient with spirometer was >0.75, which corroborates our results [14]. In our method, estimation of the thoracic region was not strict; however, we obtained a reasonable correlation by noise reduction processing. 

In addition, a difference magnitude of ±10 W between the QVT estimated by the proposed method and the VT estimated by the expiratory gas analyzer suggests that the proposed method might allow VT calculation without contact in the lamp load test using a general upright bicycle ergometer. As shown in Table 2, the difference between VE and QVE is rather large, but the trends of changes in VE and QVE are similar, as reflected by the correlation coefficient. Consequently, VT and QVT may show close values. In this experiment, because the distribution of the subjects’ VT was biased to approximately 100 W, additional tests are required for subjects with more diverse VT. However, the primary objective of this research was to construct a method to conveniently calculate the VT from an engineering perspective, which has been significantly attained in this study.

This study has some limitations. First, the sample size of this study is small, and all participants were males. Thus, it is necessary to conduct extensive studies targeting a variety of subjects from different age groups, genders, and athletic abilities. In addition, the structural and statistical examination is required for more subjects. From the perspective of motion analysis, a comparison by introducing OEP is considered to be effective for assessing the validity and reliability of the proposed method.

## 5. Conclusions

By tracking the thoracoabdominal region using the motion capture function of the Kinect v2 sensor, we proposed a non-contact respiration measurement during the exercise tolerance test corresponding to an increased body movement with increasing exercise intensity. In this study, we designed a digital filter for bandpass filter processing in real time. As a result of investigating the correlation between the QVE and the VE by simultaneous measurement using an expiration gas analyzer, a high correlation coefficient up to about 160 W of the exercise intensity was obtained. The study clarifies that posture during the pedaling motion affects the measurement accuracy of the proposed method. Nevertheless, elucidation of the condition that deteriorates the precise measurement warrants further study.

QVT estimated from QVE showed a difference of ±10 W from VT calculated by expiratory gas analyzer. In this experiment, the number of subjects, as well as difference in VT among these subjects, was small. Thus, the examination of the correlation between QVT and VT was impossible. Therefore, in this experiment, the feasibility of the proposed method could not be completely demonstrated. However, QVT estimated by the proposed method shows a value close to VT. In addition, our VT estimation method based on the knowledge of Caiozzeo shows a robust estimation accuracy in our previous research. Taken together, these results suggest that VT can be estimated by non-contact sensing for the unstable posture of the subject who pedals with an upright bicycle ergometer.

## Figures and Tables

**Table 1:** Specifications of the Kinect v2 sensor.

Item	Specification
Resolution of RGB camera	1920 × 1080 pixel
Frame rate of RGB camera	30 fps
Resolution of IR camera	512 × 422 pixel
Frame rate of IR camera	30 fps
Depth range	0.5–4.5 m
Field of view	70° (vertical), 60° (horizontal)

**Table 2:** Bias and 95% CI on the difference between the VE and the QVE, and the estimated values of VT and QVT.

Subject	Bias ± 95% CI	VT	QVT
A	−0.0092 ± 0.2290	102 W	106 W
B	0.0116 ± 0.2453	108 W	116 W
C	0.0616 ± 0.2554	112 W	102 W
D	0.1119 ± 0.3263	118 W	128 W
E	0.0455 ± 0.3035	118 W	117 W
F	0.0586 ± 0.2641	60 W	68 W
